# Proteomic Analysis of the Spinophilin Interactome in Rodent Striatum Following Psychostimulant Sensitization

**DOI:** 10.3390/proteomes6040053

**Published:** 2018-12-17

**Authors:** Darryl S. Watkins, Jason D. True, Amber L. Mosley, Anthony J. Baucum

**Affiliations:** 1 Stark Neurosciences Research Institute, Indiana University School of Medicine Medical Neuroscience Graduate Program, Indianapolis, IN 46278, USA; dswatkin@iu.edu; 2 Department of Biochemistry and Molecular Biology, Indiana University School of Medicine, Indianapolis, IN 46278, USA; Jdtrue@bsu.edu (J.D.T.); almosley@iu.edu (A.L.M.); 3 Department of Biology, Ball State University, Muncie, IN 47306, USA; 4 Department of Biology, Indiana University-Purdue University Indianapolis, Indianapolis, IN 46202, USA; 5 Stark Neurosciences Research Institute Indianapolis, Indianapolis, IN 46202, USA; 6 Department of Pharmacology and Toxicology, Indiana University School of Medicine, Indianapolis, IN 46202, USA

**Keywords:** amphetamine, spinophilin, protein phosphatase-1, dopamine, striatum

## Abstract

Glutamatergic projections from the cortex and dopaminergic projections from the substantia nigra or ventral tegmental area synapse on dendritic spines of specific GABAergic medium spiny neurons (MSNs) in the striatum. Direct pathway MSNs (dMSNs) are positively coupled to protein kinase A (PKA) signaling and activation of these neurons enhance specific motor programs whereas indirect pathway MSNs (iMSNs) are negatively coupled to PKA and inhibit competing motor programs. An imbalance in the activity of these two programs is observed following increased dopamine signaling associated with exposure to psychostimulant drugs of abuse. Alterations in MSN signaling are mediated by changes in MSN protein post-translational modifications, including phosphorylation. Whereas direct changes in specific kinases, such as PKA, regulate different effects observed in the two MSN populations, alterations in the specific activity of serine/threonine phosphatases, such as protein phosphatase 1 (PP1) are less well known. This lack of knowledge is due, in part, to unknown, cell-specific changes in PP1 targeting proteins. Spinophilin is the major PP1-targeting protein in striatal postsynaptic densities. Using proteomics and immunoblotting approaches along with a novel transgenic mouse expressing hemagglutainin (HA)-tagged spinophilin in dMSNs and iMSNs, we have uncovered cell-specific regulation of the spinophilin interactome following a sensitizing regimen of amphetamine. These data suggest regulation of spinophilin interactions in specific MSN cell types and may give novel insight into putative cell-specific, phosphatase-dependent signaling pathways associated with psychostimulants.

## 1. Introduction

Psychostimulant drug abuse is becoming increasingly popular and costly globally [1,2]. Psychostimulant drugs of abuse, such as methamphetamine, amphetamine, and cocaine, have been associated with dopamine (DA) receptor dysfunction, improper synaptic transmission, and other neuronal perturbations that may contribute to addiction pathology [3,4,5,6,7,8]. Psychostimulants drive hyper-dopaminergic signaling within the striatum by increasing DA concentrations and enhancing DA transmission [9,10,11,12,13,14]. When low doses of psychostimulants are administered chronically, response to the drug also increases, causing progressive potentiation of motor programs, a process known as behavioral sensitization [15,16]. Thus, DA plays a critical role in basal ganglia regulated motor programs [17,18,19,20,21,22].

The striatum is the largest structure within the basal ganglia and has been shown to play a role in disease states, such as Huntington and Parkinson Disease (HD and PD, respectively), and neurological disorders like obsessive-compulsive disorder (OCD) and drug addiction/abuse [23,24,25,26,27,28]. The striatum is divided into two main regions: The dorsal striatum (dStr) and the ventral striatum (vStr), which includes the nucleus accumbens (NAc) and the olfactory tubercle (OT). The dStr is innervated by dopaminergic projections arising from the substantia nigra (SN) and has been functionally described as a modulator of motor domains specifically involving action selection and initiation [29,30]. The vStr is innervated by dopaminergic projections from the ventral tegmental area (VTA) and is involved in mediating reward and motivational domains [31,32,33]. However, studies also suggest that there is significant overlap in motor and reward functional domains within the striatum [34,35]. Approximately 90–95% of the neuronal populations within the striatum are gamma-aminobutyric acid (GABA)-ergic medium spiny neurons (MSNs). There are two MSN subtypes within the striatum that are characteristically distinct based on physiological and structural properties, as well as differential expression of DA receptor subtypes and neuropeptide hormones [36,37,38]. Differential expression of DA receptors allows for differential signaling within striatal MSNs. Studies have shown that characteristics and behaviors associated with striatal specific pathological maladies can occur when there is an imbalance in the activity and/or signaling between the two MSN classes [14,39,40,41]. Direct pathway MSNs (dMSNs) contain the D1 class of DA receptors, which are positively coupled to PKA signaling. Activation of dMSNs enhances basal ganglia related motor programs. Conversely, indirect pathway MSNs (iMSNs) contain the D2 class of DA receptors, which are negatively coupled to PKA signaling. Activation of iMSNs inhibits inappropriate basal ganglia regulated motor function [42,43,44,45]. Thus, the opposing functions of dMSNs and iMSNs are, in part, regulated by post-synaptic responses to DA-dependent signaling.

In the post-synaptic density (PSD) of MSNs, reversible protein phosphorylation is facilitated by kinase and phosphatase activity, contributing to competent neuronal signaling, communication, and synaptic plasticity. To achieve proper signaling, serine/threonine kinases phosphorylate substrates utilizing specific consensus sites; however, serine/threonine phosphatases, such as protein phosphatase 1 (PP1), associate with targeting proteins to attain specificity [46,47]. The most abundant targeting protein for PP1 in the PSD is spinophilin [46,48,49,50]. Spinophilin acts as a scaffolding protein by targeting PP1 to specific substrates; however, spinophilin can also inhibit the activity of PP1, driving changes in synaptic strength and plasticity [48,51,52,53]. Furthermore, spinophilin is enriched in the PSD of dendritic spines, and is essential for proper dendritic spine function by regulation of critical dendrite properties [48,54,55,56]. Changes in MSN dendritic spine density and morphology, perturbations in synaptic transmission and concomitant aberrant dopaminergic signaling are all major contributors to striatal disease states like drug addiction and myriad others [23,26,27,31,57]. In addition, psychostimulant administration, which drives hyper-dopaminergic responses was shown to increase spinophilin expression in the striatum [58,59]. As stated above, alterations in DA levels will regulate DA receptor activity and downstream activation of kinases, such as PKA. Spinophilin is phosphorylated by PKA and PKA phosphorylation of spinophilin is known to decrease its binding to F-actin [49,60]. DA depletion, which decreases DA terminals in the striatum, modulates spinophilin interactions within the striatum. Specifically, there were increases in spinophilin binding to PP1; however, the interactions of spinophilin with a plurality of spinophilin-associated proteins (SpAPs) were decreased [61,62]. We have shown that whole-body spinophilin knockout (KO) mice do not undergo d-amphetamine-induced behavioral sensitization [63]. However, how excessive DA signaling, as occurs following psychostimulant sensitization, modulates spinophilin interactions is unclear. Here we show that in contrast to DA depletion, amphetamine sensitization increases a majority of striatal spinophilin interactions. Moreover, in our preliminary studies using a novel transgenic mouse line that allows for Cre-dependent expression of an hemagglutinin (HA)-tagged form of spinophilin, we observed both pan-MSN and putative cell-specific alterations in spinophilin interactions following amphetamine treatment. Together, these data delineate alterations in spinophilin interactions that may contribute to psychostimulant-induced pathologies.

## 2. Materials and Methods

### 2.1. Animals—HA Spinophilin Mice Generation

**Figure 1:**
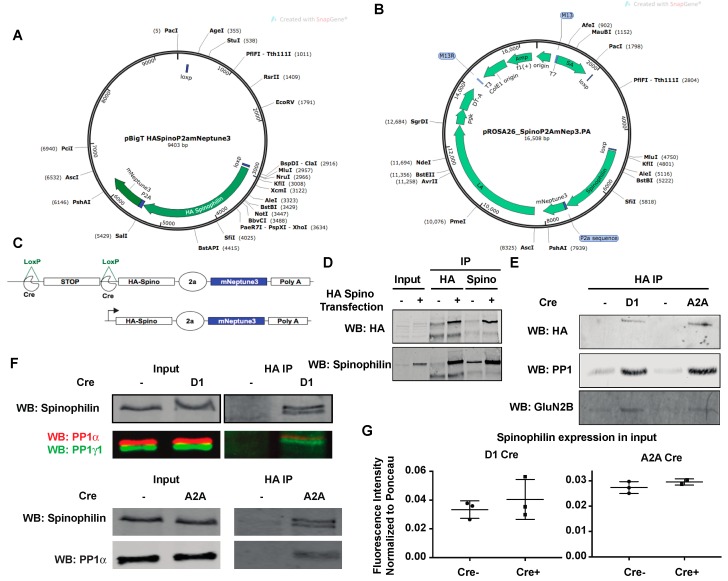
Generation and characterization of Cre-expressing, HA-tagged human spinophilin mice. (**A**) A construct containing DNA encoding HA-tagged human spinophilin with a P2A sequence and mNeptune3 fluorescent protein was cloned into the pBIGT vector that contains a floxed-stop sequence. (**B**) The construct encoding the floxed-stop sequence and the HA-spinophilin-P2A-mNeptune 3 sequence was subcloned into the ROSA targeting vector pROSA_26.PA. (**C**) The modified ROSA vector was used for generation of the targeted transgenic mice. (**D**) Striatal cells were transfected without or with HA-tagged human spinophilin. Lysates were immunoprecipitated with either an HA or spinophilin antibody and immunoblotted with an HA antibody or a spinophilin antibody. HA-spinophilin was selectively detected when it was overexpressed. (**E**) Mice express HA-tagged spinophilin upon crossing with Cre recombinase expressed in the direct pathway (D1) or indirect pathway (A2A) medium spiny neurons. (**F**) Spinophilin and protein phosphatase 1 immunoblots of inputs and HA-immunoprecipitates from HA spinophilin mice crossed with D1 or A2A Cre-recombinase-expressing mice. (**G**) Mice expressing HA-spinophilin had non-significant increases in total spinophilin expression.

All animal studies were performed in accordance with the Guide for the Care and Use of Laboratory Animals as disseminated by the U.S. National Institutes of Health and were approved by Indiana University-Purdue University School of Science Animal Care and Use Committees (Approval #SC270R). A human, HA-tagged spinophilin construct [62] containing a P2A sequence and the mNeptune 3 protein were assembled into the pBigT vector between the ClaI and SacI restriction sites (Figure 1A). Gene files were assembled in SnapGene (GSL Biotech, Chicago, IL, USA) or Vector NTI (ThermoFisher Scientific, Waltham, MA, USA). The insert was then subcloned into the AscI/PacI sites on the pROSA26.PA vector (Figure 1B) for generation of targeted embryonic stem cells (ES) cells. pROSA26.PA vector was linearized with AscI and injected into SV129 ES cells by the Vanderbilt Transgenic Mouse/ESC Shared Resource. These ES cells were transferred into pseudo-pregnant C57Bl6/J females and chimeric pups were born from two of these clones (2D4 and 2E12). Chimeras were transferred from the Vanderbilt Transgenic Mouse/ESC Shared Resource to the mouse colony at IUPUI. One clone was maintained in house. Mice were backcrossed at least six generations onto a C57Bl6/J background. Mice were subsequently crossed with either the Drd1a-Cre line or onto an A2A-Cre line [64,65] that were on the C57Bl6/J background. For proteomics, mice expressing a single copy of spinophilin knocked-in to the ROSA locus were used. For immunoblotting, mice expressing HA-spinophilin knocked into one or both copies of the ROSA locus were used. For those expressing a single copy of spinophilin, the other ROSA allele was either WT or had a flox-stop tdTomato reporter sequence inserted (Jackson laboratories Stock #007914, Bar Harbor, ME, USA).

### 2.2. Animals—Proteomics Studies

Adult male and female mice were used for the proteomics studies (Table 1).

### 2.3. Animals—Immunoblotting Studies

Four male or female P85–P120 mice were used for immunoblotting analysis of HA spinophilin. In addition, one adult P90 WT and one adult spinophilin KO mouse [63,66] were used to validate a subset of spinophilin interactions.

### 2.4. d-Amphetamine Sensitization

Mice received daily intraperitoneal (i.p) injections of d-amphetamine at 3.0 mg/kg (Sigma-Aldrich, St. Louis, MO, USA) or saline (10 mL/kg) for five consecutive days. Mice were then sacrificed and striata dissected 72 h after the last injection.

### 2.5. Brain Tissue Lysis

Mouse striatum (including both dorsal and ventral (accumbens) striatum or olfactory tubercle) was homogenized and sonicated in 1 mL in a low-ionic strength Tris buffer containing 20 mM 1M Tris, 10 mM DTT, 2 mM 0.5 M EDTA, 10% Triton X-100, 1% protease inhibitor cocktail (Bimake, Houston, TX, USA), phosphatase inhibitors (20 mM sodium fluoride, 20 mM sodium orthovanadate, 20 mM β-glycerophosphate, and 10 mM sodium pyrophosphate; Sigma-Aldrich or ThermoFisher Scientific (Waltham, MA, USA)). Homogenates were incubated for 15 min at 4 °C and then centrifuged at 13,600× *g* for 10 min. The cleared lysate was mixed with Laemmli sample buffer to generate the input or subjected to immunoprecipitation.

### 2.6. Transfections

Mouse STHdhQ7/7 striatal cell line (a kind gift from Dr. Gunnar Kwakye, Oberlin College, Oberlin, OH, USA) were cultured in Dulbecco’s modified Eagle’s medium (DMEM) that contained 10% fetal bovine serum, 1% GlutaMAX^TM^ (ThermoFisher Scientific), 400 μg/mL G418-Sulfate (Geneticin) (ThermoFisher Scientific), 100 U/mL penicillin, and 100 μg/mL streptomycin. Culture plates were incubated at a constant 33 °C and 5% CO_2_ in myTemp Mini CO_2_ digital incubator (Benchmark Scientific; Edison, NJ, USA). Cells were transfected overnight with 2 μg of HA-tagged human spinophilin and PolyJet reagent (SignaGen Laboratories, Gaithersburg, MD, USA) per the manufacturers’ instructions. Cells were lysed in the low-ionic strength Tris buffer.

### 2.7. Immunoprecipitations

Striatal lysates were immunoprecipitated with an HA-epitope antibody or spinophilin antibody. 3 µg of goat HA polyclonal antibody (Bethyl Laboratories, Montgomery, TX, USA, A190-238A) or 5 µg goat spinophilin polyclonal antibody (Santa Cruz Biotechnology, Dallas, TX, USA, SC14774) were incubated at 4 °C with 750–800 µL (75–80%) of total striatal lysate overnight. Striatal cell lysates were immunoprecipitated with 1.6 µg of a sheep spinophilin antibody (ThermoFisher Scientific). The following day, protein G magnetic beads (DynaBeads, ThermoFisher Scientific) were added, and the mixture was incubated for 2 h. Beads were washed three times by magnetic separation in an immunoprecipitation wash buffer (50 mM NaCl, 50 mM Tris-HCl pH 7.5, 0.5% (*v*/*v*) Triton X-100). For immunoblotting, beads were resuspended in 2X sample buffer. For Tandem Mass Tag (TMT) labeling, beads were subsequently washed three times in PBS by centrifugation. Washed beads were submitted for tryptic digestion and each sample was labeled with an isobaric tandem mass tag to allow for quantitation.

### 2.8. TMT Labeling and Mass Spectrometry

Following washes, immunoprecipitated samples on beads were reduced with 5 mM tris(2-carboxyethyl)phosphine hydrochloride (TCEP) and alkylated with 10 mM chloroacetamide (CAM). Beads were then incubated with Trypsin Gold (Promega) at 37 °C overnight. Digested samples were cleaned up using a Waters Sep-Pak C18 plate per manufacturer’s instructions.

For TMT labeling, tryptic peptides from each individual condition were labeled with eight different isobaric TMT tags using 8 of a 10-plex TMT kit (ThermoFisherScientific) and following manufacturer’s instructions. Following individual labeling, samples were mixed and separated by HPLC and subjected to mass spectrometry.

For HPLC and mass spectrometry analysis, digested peptides were loaded onto an Acclaim PepMap C18 trapping column and eluted on a PepMap C18 analytical column with a linear gradient from 3% to 35% acetonitrile (in water with 0.1% formic acid) over 120 min in-line with an Orbitrap Fusion Lumos Mass Spectrometer (ThermoFisher Scientific). Raw files generated from the run were analyzed using Thermo Proteome Discoverer (PD) 2.2. SEQUEST HT (as a node in PD 2.2) was utilized to perform database searches as previously described [67] with a few modifications: Trypsin digestion, two maximum missed cleavages, precursor mass tolerance of 10 ppm, fragment mass tolerance of 0.8 Da, fixed modifications of +57.021 Da on cysteine, +229.163 on lysines and peptide N-terminii, and a variable modification of +15.995 Da on methionine. The spectral false discovery rate (FDR) was set to ≤1% as previously described [68]. The FASTA database used was a mouse proteome downloaded from Uniprot on January 9, 2017 with the addition of 72 common contaminants. Results and quantitative information from the TMT were exported to an Excel Spreadsheet (Tables S1 and S2, Microsoft, Seattle, WA, USA) and tables were generated from these data. A total intensity from the TMT labels, derived from all of the tryptic peptides matching to a specific protein, is given. These intensity data were used for comparing the abundance of different proteins. Examples of these TMT peaks are shown in Figures S2–S5.

### 2.9. Immunoblotting

Striatal lysates were immunoblotted with the appropriate primary antibody (see below). For protein detection the following primary antibodies were used: HA rabbit antibody (Bethyl Laboratories A190-208A), mouse Clathrin Heavy Chain monoclonal antibody (Santa Cruz, CA, USA, sc12734), rabbit SAP102 monoclonal antibody (Cell Signaling Technology, Danvers, MA, USA; 47421S), rabbit SNIP/p140Cap (SRCIN1) polyclonal antibody (Cell Signaling Technology 3757) and goat or sheep spinophilin polyclonal antibody (as above) or rabbit spinophilin antibody (Cell Signaling Technology 14136S). Antibody dilutions were used at 1:500–1:2000 for immunoblotting. Following overnight incubation at 4 °C, the following secondary antibodies were used for fluorescence detection: Donkey anti-rabbit (H+L) Alexa Fluor 790 (Jackson Immunoresearch, West Grove, PA, USA #711-655-152, 1:50,000 dilution), Donkey anti Goat Alexa Fluor 680 (ThermoFisher Scientific A21084; 1:10,000 dilution), Donkey anti-mouse (H+L) Alexa Fluor 680 (ThermoFisher Scientific, A10038; 1:10,000 dilution), Donkey anti-mouse (H+L) Alexa Fluor 790 (Jackson Immunoresearch #715-655-151, 1:50,000). Imaging was performed on an Odyssey CLx system (LI-COR Biosciences, Lincoln, NE, USA).

### 2.10. Pathway Analysis

Proteins that were increased in HA-spinophilin immunoprecipitates isolated from amphetamine treated animals were analyzed using the Database for Annotation, Visualization and Integrated Discovery (DAVID) Bioinformatics Resource (version 6.8; https://david.ncifcrf.gov/, National Cancer Instutitue at Frederick, MD, USA [69,70]). Proteins were analyzed using the Kyoto encyclopedia of genes and genomes (KEGG) and gene ontology (GO) pathway databases.

### 2.11. Statistics

To compare saline to amphetamine treatment across all groups combined, a *t*-test was performed to compare spinophilin abundance in the amphetamine vs. saline treated samples. A non-adjusted *t*-test and a *t*-test adjusted for multiple comparisons (using the Holm-Sidak method) were performed to compare the abundance of the proteins isolated from amphetamine compared to saline-treated samples (both non-normalized (Table S1) and normalized (Table S2). For normalization for PCA analysis and protein abundance, we divide the abundance of the individual protein in the individual sample by the total peptide or total spinophilin abundance detected in that sample. Given that there was an N of 1 in some of the sub-categories (e.g., A2A male and D1 female) the study was not powered nor intended to make statistical conclusions and these results are a qualitative display of sex- and cell-specific protein interactions.

## 3. Results

### 3.1. Generation of HA-Tagged Spinophilin Mice

We created constructs that encoded HA-tagged human spinophilin [62] along with a P2A sequence and a far-red fluorescent protein (mNeptune3 [71]). Mice were generated from these constructs by the Vanderbilt Transgenic Mouse/ESC Shared Resource (see methods). When crossed with Cre-expressing mice, these animals express HA-tagged human spinophilin under control of the ROSA promoter (Figure 1C). When crossed with mice expressing Cre recombinase under control of the *Drd1a* gene or the *Adora2a* gene, we were able to detect HA signal in HA immunoprecipitates by immunoblotting (Figure 1D,E). Furthermore, we were able to detect known spinophilin interacting proteins PP1 and GluN2B [48,50,72,73] in the HA immunoprecipitates isolated from Cre-expressing lines (Figure 1E). Less PP1 and GluN2B co-precipitated from the Cre-negative animals (Figure 1E). Moreover, a spinophilin antibody also detected a band in the HA immunoprecipitates isolated from Cre-expressing, but not Cre-negative mice (Figure 1D,F). Of note, we detected a doublet in the HA-immunoprecipitates when immunoblotting for spinophilin isolated from the HA-spinophilin expressing mice, as well as a striatal cell line transfected with an HA-spinophilin construct (Figure 1F). This is not surprising as spinophilin is thought to homo-dimerize and this suggests that the human HA-spinophilin is complexing with the endogenous, mouse spinophilin. However, there was no significant difference in the amount of total spinophilin in the mice expressing HA-spinophilin, suggesting a low overexpression of spinophilin (Figure 1G). Moreover, given the low expression of epitope tagged spinophilin and fluorescent protein, we were unable to detect either HA-tagged protein or fluorescent protein by immunohistochemistry (data not shown).

### 3.2. Amphetamine Modulates Spinophilin Expression and Interactions

**Figure 2:**
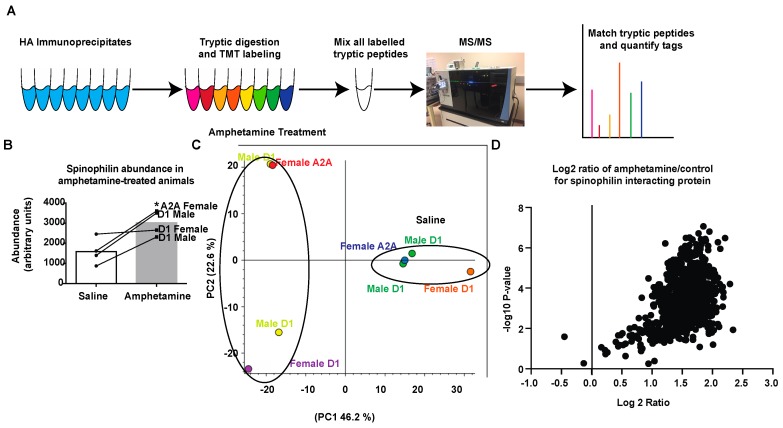
Quantitation of spinophilin complexes isolated from dMSNs and iMSNs using tandem mass tag (TMT) analysis. (**A**) Striatal lysates isolated from male or female mice expressing HA spinophilin under the control of D1 or A2A promoters and treated with saline or amphetamine were immunoprecipitated with an HA antibody, digested with trypsin, labelled with eight different TMT tags, mixed and analyzed by mass spectrometry (MS/MS). (**B**) A higher intensity of TMT reporter abundance matching spinophilin was observed in amphetamine-treated compared to saline treated animals (*t*-test; * *p* < 0.05). (**C**) Principal component analysis of individual samples normalized to total peptide amount within each sample. (**D**) A volcano plot showing a majority of the protein have increased abundance in HA immunoprecipitates isolated from amphetamine treated animals.

DA signaling within the striatum modulates MSN activity and signaling [74,75]. Amphetamine increases the release of DA at dopaminergic terminals synapsing on MSNs [9,11,12]. Our previous studies show that DA depletion alters the spinophilin interactome [62] and that spinophilin KO mice do not undergo amphetamine-induced locomotor sensitization [63]. However, how spinophilin normally contributes to synaptic changes associated with amphetamine-dependent striatal changes is unclear. As spinophilin targets PP1 to regulate synaptic protein phosphorylation, in order to identify potential spinophilin-dependent synaptic protein targets that are regulated by spinophilin following amphetamine sensitization, we utilized our HA-tagged spinophilin mice (Figure 1) to measure spinophilin interactions in saline- or amphetamine-treated mice expressing spinophilin in D1 DA or A2A adenosine-receptor containing neurons of the striatum. Mice were injected with 3 mg/kg amphetamine every day for five days and sacrificed 72 h after the final amphetamine treatment. Striatal lysates were immunoprecipitated with an HA antibody, digested with trypsin, labeled with TMTs, and analyzed by mass spectrometry (Figure 2A). A total TMT abundance for spinophilin was detected in all conditions. As human and mouse spinophilin differ by fewer than 30 amino acids (Figure S1) and as spinophilin homo-dimerizes we searched only the mouse database. Forty-eight spectral counts matching spinophilin were detected across all eight samples. While the human construct and mouse spinophilin are highly homologous, there are 28 (out of 817) different amino acids between the two species that lead to the generation of ~12 different potential tryptic fragments (Figure S1). We validated two MS/MS spectra generated from tryptic peptides that were predicted to be different between mouse and human spinophilin (Figures S2–S5). This further validates the expression of our HA-tagged human spinophilin construct. Based on the unnormalized abundance of the TMT tag from the different samples, we observed more spinophilin in the amphetamine-treated compared to control treated samples (Figure 2B). A principal component analysis from all proteins (1454) of the individual samples, normalized to total peptide amount, revealed that 46.2% of the total variability is due to amphetamine treatment (Figure 2C). We next evaluated changes in the TMT tag abundance ratios (amphetamine/saline) of the spinophilin interacting proteins. For this, we eliminated all contaminant proteins and only included those proteins detected in all eight samples (e.g., with all eight tags). This led to the detection of 984 total proteins in the HA-spinophilin immunoprecipitates (Table S1). We plotted these unnormalized values using a volcano plot with Log2 abundance ratio on the X-axis and −log10 *p*-value (*t*-test, non-adjusted) on the Y-axis (Figure 2D). All but two proteins showed an increased abundance in spinophilin immunoprecipitates isolated from amphetamine compared to saline treated samples.

### 3.3. Regulation of Spinophilin Interactions by Amphetamine

**Figure 3:**
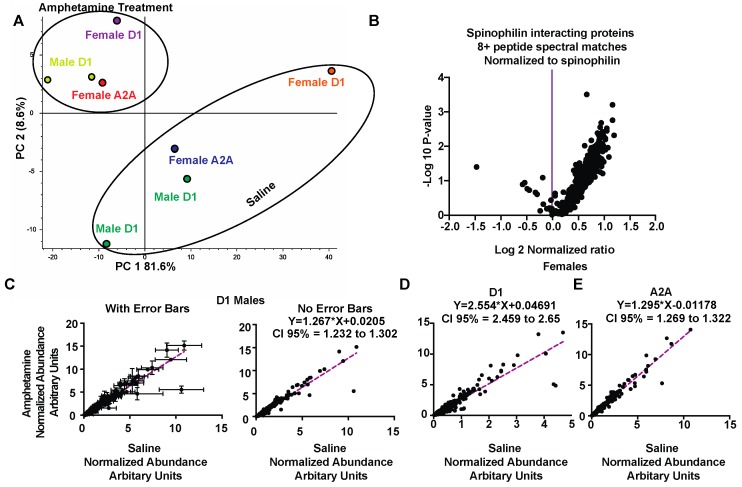
Greater abundance of spinophilin interacting proteins in amphetamine-treated animals occurs across both sexes and cell types. (**A**) Principal component analysis of individual samples normalized to spinophilin abundance within each sample and filtered for eight or more PSMs. (**B**) A volcano plot showing a majority of the proteins have increased abundance in HA immunoprecipitates isolated from amphetamine treated animals when normalized to the amphetamine-dependent increase in spinophilin abundance. (**C**) A plot of the abundance of spinophilin interacting proteins isolated from male, D1 Cre expressing animals and normalized for spinophilin expression (Table S2) and quantified from treated (Y-axis) or control (X-axis) samples. Left panel shows mean ± standard deviation, the right panel just shows the mean of the two values. (**D**) A plot of the abundance of spinophilin interacting proteins isolated from female, D1 Cre expressing animals and normalized for spinophilin expression (Table S2) and quantified from treated (Y-axis) or control (X-axis) samples. (**E**) A plot of the abundance of spinophilin interacting proteins isolated from female, A2A-Cre expressing animals and normalized for spinophilin expression (Table S2) and quantified from treated (Y-axis) or control (X-axis) samples.

Given that spinophilin abundance was increased in immunoprecipitates isolated from amphetamine treatment compared to saline-treated samples, to determine if the increased association of spinophilin with interacting proteins was due exclusively to increased spinophilin levels, we normalized the abundance of each individual interacting protein to the abundance of spinophilin in the corresponding sample (Table S2A). Moreover, in Table S2, we show only those proteins that contained at least eight peptide spectral matches (PSMs) as these would average 2 PSMs per condition. We detected 423 total proteins across all conditions that met these criteria. Of these proteins, 134 were unchanged (Log2 ratio −0.5 to +0.5), three had a decreased association (<Log2 Ratio −0.5), and 286 had an increased association (>Log2 Ratio +0.5) with spinophilin. Those proteins with a decreased interaction ratio of <−0.5 and increased interaction ratio of >1.0 are shown in Table 2. We performed a second PCA analysis of the data normalized to spinophilin for those peptides having eight PSMs or more (Figure 3A). This mode of analysis decreased the variability within the amphetamine treatment group but increased the variability in the saline treatment group. We plotted these normalized values using a volcano plot with Log2 abundance ratio on the X-axis and −log10 P-value (*t*-test, non-adjusted) on the Y-axis (Figure 3B). Therefore, even when normalized to spinophilin there is a higher number of proteins with an enhanced association with spinophilin compared to a decreased or no change in association.

We next evaluated the spinophilin interacting proteins from the different cell types and sexes. It is important to note that evaluation of these sub-categories (sex and genotype) are qualitative and no statistical inference can be made; however, these preliminary studies denote the importance of evaluating different sexes and cell types. We plotted the abundance of the amphetamine treated samples on the Y-axis and the abundance of the saline-treated samples isolated from D1 males (Figure 3C), D1 females (Figure 3D) and A2A females (Figure 3E). All data were normalized to spinophilin abundance. All three sets had a slope greater than 1, suggesting that there was greater abundance in the amphetamine treatment compared to the saline treatment. Of note, the D1 females had the greatest slope (M = 2.554), suggesting the greatest increased association in this group compared to the D1 males (M = 1.267) or A2A females (M = 1.269). Together, our data suggest that amphetamine treatment enhances synaptic protein interactions with spinophilin across multiple sexes and cell types.

To begin to delineate amphetamine-dependent regulation of specific spinophilin interactors in the different Cre lines, we generated a ratio of the abundance ratios from the D1 Cre animals to the A2A Cre animals (Table S2B). As stated above, it is important to note that, given the N of 1 in the A2A animals, these ratios are qualitative and no statistical inference can be obtained; however, these data will inform novel lines of inquiry in future cell-specific studies. Those proteins with this ratio of ratios greater than 2 are shown in Table 3. No interactions with a ratio of less than 0.5 (2-fold decrease) were detected. All ratios are shown in Table S2B.

### 3.4. Pathway and GO Analysis of Spinophilin Interacting Proteins Enhanced by Amphetamine

Using the DAVID Bioinformatics resource [69,70], we performed the Kyoto Encyclopedia of Genes and Genomes (KEGG) pathway analysis on the 286 proteins that had an increased association with spinophilin across all samples. 283 total proteins were detected from the list. A total of 94 pathways were detected from this analysis (Table S3). 49 of the pathways were significantly enriched using a Bonferroni adjustment (Table S3 highlighted). These include pathways associated with striatal function, including amphetamine addiction. The top 10 pathways also include other disease states associated with striatal dysfunction, such as Parkinson disease and Huntington disease (Table 4).

We next evaluated these increased interactions in DAVID using gene ontology terms (GO). We evaluated Biological Processes (BP), Cellular Components (CC), and Molecular Function (MF). We detected 247 BPs, 154 CCs, and 127 different MFs (Table S4) in the increased spinophilin interactors. Of these, 33, 55, and 31, respectively, were significantly enriched. For BPs, we observed a large number of metabolic processes, including ATP and NADH metabolism. We also observed vesicle trafficking processes, including synaptic vesicle endocytosis, vesicle-mediated transport, and endocytosis. For CCs, we observed known areas where spinophilin is enriched, including membrane, postsynaptic density, dendrite, dendritic spine, and cytoskeleton. In addition, we matched other locations where spinophilin has been implicated, such as synaptic vesicle membrane [76]. For MFs, we observed known roles for spinophilin as a scaffold, including protein binding, protein complex binding, protein kinase binding, and actin filament binding. In addition, we observed novel putative roles for spinophilin, including GTPase binding, ATP binding, and syntaxin-1 binding. The top 10 pathways for BP, CC, and MF are shown in Table 5.

### 3.5. Interactome Analysis of Spinophilin Interacting Proteins Enhanced by Amphetamine

**Figure 4:**
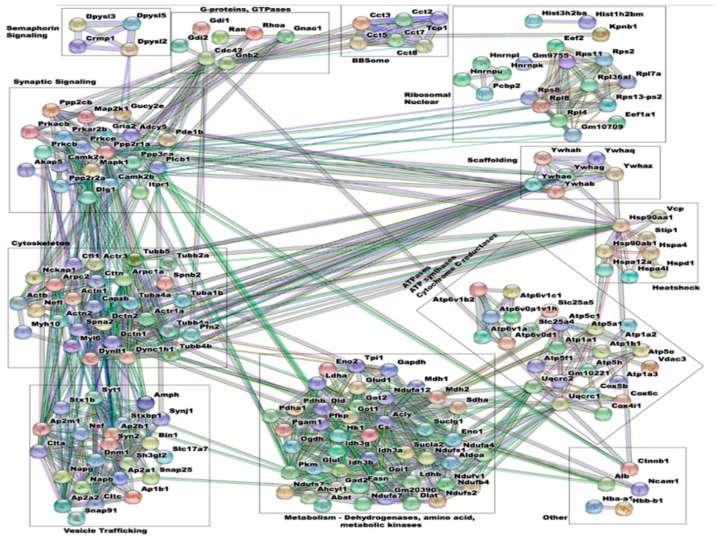
Graphical representation of interactors input into the string-db from protein complexes that had greater abundance in the HA immunoprecipitates isolated from amphetamine-treated animals. Proteins with greater abundance in HA immunoprecipitates isolated from amphetamine-treated animals were input into the string-db program (www.string-db.org) and separated by hand based on function. To reduce the complexity of this map, only those proteins that had at least 1 interaction at a confidence of 0.9 (highest confidence).

We next wanted to organize spinophilin interacting proteins that were enhanced by amphetamine based on interactions and functional classifications. To do this, we utilized the string-db program [77,78]. This allows for the pictorial representation of proteins and their interactions. To reduce the complexity of the submitted proteins, we used a high stringency confidence score (0.900) and removed any proteins that were not connected. We next grouped proteins into 12 categories based on known function and these interactions (Figure 4). These categories are: Metabolism, ATPases, vesicle trafficking, synaptic signaling, cytoskeleton, ribosomal and nuclear, scaffolding, heatshock, G-proteins and GTPases, semaphorin signaling, BBSome, and other.

### 3.6. Alteration and Validation of Novel Spinophilin Interacting Proteins

**Figure 5:**
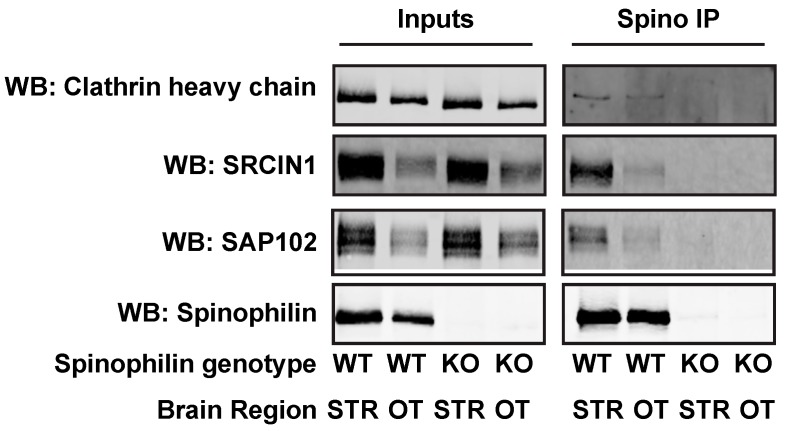
Validation of spinophilin interactions. WT or spinophilin KO striatal (STR) or olfactory tubercle (OT) lysates were immunoprecipitated with a spinophilin antibody. Lysates or immunoprecipitates were immunoblotted for spinophilin and three interacting proteins that were detected in the HA immunoprecipitates that had a decreased (SAP102) or increased (Clathrin heavy chain and SRCIN1) interaction with spinophilin in amphetamine-treated animals. Spinophilin and all associated proteins were detected in the spinophilin immunoprecipitates from WT animals, but were absent in immunoprecipitates isolated from KO animals.

While some of the proteins isolated using the HA spinophilin antibody are known spinophilin interactors (e.g., PP1, glutamate receptors, actin), some have not been previously validated. Therefore, we wanted to validate some of the proteins that had an altered association with spinophilin that are involved in different processes. We chose SAP102 (*Dlg3*), src kinase inhibitor protein 1 (*Srcin1*), and clathrin heavy chain (*Cltc*) as proteins involved in synaptic scaffolding, signaling, and vesicle trafficking, respectively. To determine if these proteins interact with spinophilin in a specific manner, we dissected out total (dorsal and ventral (e.g., accumbens)) striatum (Str) and olfactory tubercle (OT), a further ventral portion of the striatum from wildtype and whole-body spinophilin KO animals. We chose these regions based on their roles in psychostimulant sensitization [31,32,33]. We immunoprecipitated striatal and tubercle lysates with a spinophilin antibody and immunoblotted for spinophilin, SAP102, Srcin1, and clathrin heavy chain. Spinophilin was only detected in WT and not KO samples (Figure 5). Moreover, spinophilin interactions were only detected in WT and not KO animals. These data suggest that these proteins are specific interactors with spinophilin.

## 4. Discussion

### 4.1. Spinophilin Functional Localization

Spinophilin is a highly abundant spine-enriched protein. We have previously found that loss of spinophilin modulates striatal behaviors. Specifically, loss of spinophilin decreases motor performance and motor learning on a rotarod apparatus [66]. Moreover, we previously reported that spinophilin KO mice, in contrast to WT mice, do not undergo amphetamine-induced locomotor sensitization [63]. In addition, others have observed alterations in the response of spinophilin KO mice in rotarod behaviors and following cocaine treatment [79,80]. Together, these data suggest that spinophilin is important in striatal based behaviors. However, how spinophilin contributes to these behaviors is unclear. As the major postsynaptic density-enriched PP1-interacting protein, spinophilin’s functional regulation of the above striatal behaviors may be due to its targeting of PP1 to synaptic proteins. Moreover, while spinophilin, as its name implies, is enriched in dendritic spines, it is also present in dendrites, presynaptic terminals, and glial cells [54,55]. Therefore, spinophilin may have functions beyond just dendritic spines.

### 4.2. Regulation of the Spinophilin Interactome Following Amphetamine Treatment

To begin to identify cell-type specific spinophilin interactions that may be important in behavioral changes observed following psychostimulant abuse, we created mice that Cre-dependently overexpress an epitope-tagged (HA) form of human spinophilin. Using HA immunoprecipitation from different cell types and TMT labeling, we probed the spinophilin interactome in the striatum of saline compared to amphetamine treated animals. As previously observed in rats [58,59], the amount of spinophilin detected (based on labeled peptide abundance) was increased following amphetamine treatment. Interestingly, across all samples the abundance of the different proteins was greater in the amphetamine-treated compared to the control treated lysates, even when normalizing to the increased spinophilin expression. This is in contrast to what we observed previously in DA-depleted striatum (an animal model of Parkinson disease) [62]. In that previous study, 60 proteins were decreased whereas 31 total proteins were increased across two different fractions. In contrast, in the current study, we only observed three proteins that were decreased and 286 proteins that were increased following amphetamine treatment. These data suggest that spinophilin interactions are decreased by DA depletion and increased by hyperdopaminergic signaling. However, it is critical to note that while specific, there are low levels of expression of the HA-tagged, human spinophilin and while we validated expression of the HA-tagged form of the human protein by WB and MS/MS, as well as the interactor, PP1, additional interacting proteins may be non-specifically interacting with the beads. Therefore, future studies will need to follow-up on these studies to delineate those interactions that are real and that are modulated by amphetamine.

Regulation of DA leads to alterations in striatal medium spiny neuron spine density. Specifically, loss of DA decreases spine density [81,82,83] and psychostimulant treatment increases spine density [84,85]. Spinophilin is also known to regulate spine density, with acute knockdown of spinophilin decreasing spine density in hippocampal cultures [86]. Whole-body spinophilin KO animals do not have loss of dendritic spines in adulthood (and have a paradoxical increase in young animals) [56]. This lack of an effect may be due to compensatory changes, such as decreases in expression of PP1 [80]. However, data showing amphetamine-dependent increases in spinophilin expression [58,59] and our data showing a lack of amphetamine-dependent locomotor sensitization [66] and the data presented in this paper may suggest that changes in the spinophilin expression and/or interactions are critical for normal psychostimulant-induced behaviors. However, it is currently unclear if amphetamine-induced increases in spine density are also abrogated or modulated in spinophilin KO mice.

### 4.3. Classes and Specific Spinophilin Protein Interactions that are Modulated by Amphetamine

Using GO and KEGG analysis along with hand annotation of altered interactions in string-db we detailed different classes of spinophilin interacting proteins that have an increased interaction with amphetamine. These protein classes co-purify with spinophilin, including cytoskeletal and vesicle trafficking proteins [62,73,87,88]. Moreover, many of the pathways identified associate with protein binding, striatal function and diseases, and synaptic/postsynaptic protein organization. In addition to these known functions of spinophilin and striatum-dependent regulation by amphetamine, we observed novel/less well-characterized spinophilin interactions. Some of these may be non-specific. For instance, myelin sheath was one of the most abundant cellular components. Myelin sheath components may be non-specifically sticky and may not be specific interactions. Indeed, myelin sheath components are associated with the CRAPome [89], a list of non-specific interactions that may non-specifically co-precipitate. However, even though some of these interactions may be non-specific, these pathways may be regulated by amphetamines. For instance, psychostimulant abuse in humans may be associated with altered neuron myelination [90]. Therefore, while additional studies will need to detail specific spinophilin interactions, our data may delineate alterations in protein expression following amphetamine treatment.

Another major class of altered spinophilin interacting proteins were vesicle trafficking proteins. For instance, the synaptic vesicle cycle was identified as being enriched in the KEGG pathway and we delineated multiple vesicle trafficking proteins. While not much is known about the role of spinophilin in vesicle trafficking, one study has detailed spinophilin as a regulator of presynaptic vesicle function [76]. We also validated a vesicle trafficking protein, clathrin heavy chain, as a specific interactor with spinophilin. Spinophilin may play a role in vesicle trafficking on glutamatergic (or dopaminergic) presynaptic terminals or postsynaptic MSNs; however, given the enrichment of endogenous spinophilin in spines and a lack of detected presynaptic vesicle proteins (e.g., syntaxin, SNAP-25, synaptobrevin, etc.), we posit that this role is more postsynaptic; however, we cannot rule out a presynaptic role for spinophilin in striatum.

We observed many metabolic proteins, including lactate and succinate dehydrogenases, and ATPases, that were increased in spinophilin immunoprecipitates following amphetamine treatment. Moreover, some of the pathways with altered protein expression included glycolytic process, ATP metabolism/hydrolysis/synthesis, tricarboxylic acid cycle, and Citrate (TCA) cycle. Spinophilin has been shown to associate with and regulate the membrane localization of the Na^+^-K^+^-ATPase [91]. Moreover, amphetamines may modify the activity of the Kreb’s cycle [92,93]. However, how alterations in spinophilin interactions with metabolic proteins modulate response to amphetamines is an unexplored area.

### 4.4. Direct and Indirect Pathway Striatal MSNs and Spinophilin Interactions

By using mice expressing spinophilin Cre dependently, we were able to isolate complexes from dMSNs and iMSNs. We used a D1 DA receptor Cre line and an A2A adenosine receptor Cre line. These Cre lines were created as part of the GENSAT project [64,65]. While D1 and A2A are enriched in striatal MSNs, there may be expression in other cell types and other brain regions. We observed some HA spinophilin expression in other brain regions, including the hippocampus and prefrontal cortex (data not shown). While these Cre lines only minimally express in these other regions, it may be sufficient for driving expression of the HA spinophilin.

Previous studies have observed persistent psychostimulant-dependent increases in spinophilin density in dMSNs (months), whereas in iMSNs these changes are more transient (days) [84]. We found that there was a greater association of spinophilin with multiple proteins 72 h following amphetamine treatment and that this occurred in both Cre-driver lines. This suggests that amphetamine may be regulating interactions in both cell types. While our preliminary study suggests the level of increase was greater overall in the dMSNs compared to the iMSNs, it is unclear if these changes will persist in both populations and future studies will need to evaluate the persistence of these changes and the link between spinophilin and modulation of dendritic spine density.

While most protein interaction changes were similar in the two cell types, the magnitude of the effect was different between the different cell types. However, there was a cell-specific effect of amphetamine treatment in two proteins that are known to be involved in striatal pathologies. We detected both α-synuclein (*SNCA*) and tau (*MAPT*) proteins in the HA-spinophilin immunoprecipitates. While additional work needs to determine if endogenous spinophilin associates with these proteins, it was interesting that amphetamine increased the association of spinophilin with both of these proteins, but this increase was only in the D1 Cre containing animals. These proteins were enriched 2.45-fold (α-synuclein) and 1.85-fold (Tau) in the dMSNs compared to the iMSNs. These proteins play major roles in PD and AD, respectively, and amphetamine is known to increase α-synuclein and tau protein levels [94,95]. Moreover, phosphorylation of these proteins is important in modulating their function and aggregation potential, but if spinophilin plays a role in modulating this aggregation has, to our knowledge, not been evaluated.

## 5. Conclusions

Our data identify novel putative spinophilin interactions that are modulated by amphetamine. As a whole, amphetamine increased spinophilin expression and enhanced spinophilin interactions. While these changes occur in both MSN cell types, this preliminary study suggests dMSNs appear to be more influenced by amphetamine. Future studies need to validate these interactions, use additional approaches to enhance cell specific expression and interactions (e.g., viral transduction of Cre-dependent epitope tagged spinophilin), and evaluate long-term amphetamine changes (e.g., 1-month) in the different striatal MSN subtypes. However, the current proteomics study is the first to outline potential pathways of spinophilin interactors that are modulated by amphetamine. Moreover, we have begun to uncover differences in amphetamine-dependent spinophilin interactions in the different striatal cell types. This knowledge will enhance our understanding of amphetamine-dependent regulation of cell-specific striatal biology.

## Figures and Tables

**Table 1:** Animals used for proteomics studies. Sex, genotype, weight of animals used for proteomics studies.

Eartag	Sex	Condition	Genotype	Cre	Initial Weight	Final Weight	Birth Date	Sacrifice Date
2450	M	Saline	Het/Cre+	D1	23.8	25.0	31 January 2018	30 March 2018
2452	M	Treated	Het/Cre+	D1	22.4	23	31 January 2018	30 March 2018
2453	M	Treated	tdHet/HA-Het/Cre+	D1	23.0	23.3	31 January 2018	30 March 2018
2454	M	Saline	Het/Cre+	D1	22.3	22.8	31 January 2018	30 March 2018
2390	F	Saline	Het/Cre+	A2A	22.9	22.8	3 January 2018	30 March 2018
2393	F	Treated	Het/Cre+	A2A	20.9	20.8	3 January 2018	30 March 2018
2443	F	Saline	Het/Cre+	D1	21.5	21.7	29 January 2018	30 March 2018
2444	F	Treated	Het/Cre+	D1	19.6	19.8	29 January 2018	30 March 2018

**Table 2:** Spinophilin interacting proteins that had altered abundance following amphetamine treatment.

Description	# PSMs	Normalized Abundance Ratio (Treatment)/(Control)	Normalized Abundance Ratio (log2): (Treatment)/(Control)
**Decreased Interactions**			
E3 ubiquitin-protein ligase XIAP	22	0.69	−0.54
Disks large homolog 3	143	0.67	−0.58
Granulins	10	0.36	−1.47
**Increased Interactions**			
Myelin proteolipid protein	38	2.28	1.19
Hemoglobin subunit alpha	17	2.24	1.16
ADP/ATP translocase 2	36	2.23	1.16
Clathrin light chain A	17	2.13	1.09
Adenylyl cyclase-associated protein 2	8	2.10	1.07
MCG10343, isoform CRA_b	35	2.08	1.05
Tubulin alpha-1B chain	54	2.07	1.05
Tubulin alpha chain (Fragment)	54	2.03	1.02
Cytochrome c oxidase subunit NDUFA4	13	2.03	1.02
Profilin-2	14	2.02	1.02
Reticulon (Fragment)	20	2.02	1.01
Myelin-oligodendrocyte glycoprotein	18	2.01	1.01
Serine/threonine-protein phosphatase 2A 65 kDa regulatory subunit A alpha isoform	28	2.00	1.00

HA spinophilin was immunoprecipitated from saline and amphetamine-treated D1 Cre and A2A Cre mice. The abundance of the individual proteins was normalized to the abundance of spinophilin. A ratio of the abundance of proteins isolated from the amphetamine treated over the saline treated mice was generated. A subset of spinophilin interacting proteins that had at least eight spectral counts (PSMs) and had a decreased (<0.05) or increased (≥1.00) log2 ratio is shown. A complete list of interacting proteins (without contaminants) and their abundance ratios are shown in Table S2A.

**Table 3:** Spinophilin interacting proteins that had altered abundance ratios in D1 Cre animals compared to A2A Cre animals.

Description	PSMs	Female A2A Ratios	Female D1 Ratios	Male D1 Avg Ratios	Avg D1/A2A Ratios
Endophilin-A2	12	0.50	1.38	1.28	2.63
Cofilin-1	14	1.13	4.14	1.57	2.52
Malate dehydrogenase, mitochondrial	27	0.92	3.22	1.38	2.51
Calreticulin	8	0.87	3.15	1.11	2.46
Alpha-synuclein	21	1.06	3.56	1.61	2.43
Malate dehydrogenase, cytoplasmic	41	1.05	3.59	1.38	2.36
Glutamate dehydrogenase 1, mitochondrial	23	1.05	3.58	1.39	2.36
Fructose-bisphosphate aldolase	87	1.14	3.93	1.41	2.35
Protein disulfide-isomerase A3	9	0.93	3.19	1.13	2.31
Citrate synthase, mitochondrial	32	1.37	4.58	1.55	2.24
Synapsin-1	42	1.11	3.51	1.45	2.23
Tubulin polymerization-promoting protein	9	1.08	3.24	1.56	2.23
Nucleoside diphosphate kinase	12	1.20	3.92	1.39	2.21
Fascin	26	0.89	2.78	1.13	2.20
Cytochrome b-c1 complex subunit 1, mitochondrial	19	0.99	3.09	1.26	2.19
Carbonic anhydrase 2	14	1.22	3.75	1.54	2.18
Rab GDP dissociation inhibitor alpha	22	1.12	3.58	1.26	2.16
Protein kinase C and casein kinase substrate in neurons protein 1	18	1.10	3.36	1.30	2.12
Endophilin-A1	23	1.11	3.37	1.33	2.12
Cytochrome c oxidase subunit 5B, mitochondrial	13	1.17	3.52	1.43	2.12
Adenylyl cyclase-associated protein 2	8	1.43	4.36	1.63	2.09
Cytochrome b-c1 complex subunit 2, mitochondrial	40	1.04	3.08	1.22	2.06
Pyruvate kinase PKM	36	1.10	3.22	1.30	2.06
Profilin-2	14	1.33	3.85	1.58	2.04
Myelin proteolipid protein	38	1.58	4.56	1.89	2.04
60S ribosomal protein L17	8	1.06	3.20	1.12	2.03
40S ribosomal protein S23	11	0.89	2.40	1.23	2.03
Beta-synuclein	16	1.18	3.31	1.47	2.03
Heat shock 70 kDa protein 4	23	1.07	3.01	1.27	2.01
L-lactate dehydrogenase B chain	28	1.23	3.58	1.35	2.00

HA spinophilin was immunoprecipitated from saline and amphetamine-treated D1 Cre and A2A Cre mice. The abundance of the individual proteins was normalized to the abundance of spinophilin. A ratio of the abundance of proteins isolated from the amphetamine treated over the saline treated mice was generated. A second ratio comparing the amphetamine/saline ratios identified in the 3 D1 samples and the 1 A2A sample was generated. Those D1/A2A ratios ≥ 2.00 are shown. A complete list of interacting proteins (without contaminants) and the cell-specific abundance ratios are shown in Table S2B.

**Table 4:** Top 10 Kyoto Encyclopedia of Genes and Genomes (KEGG) pathways associated with proteins that have amphetamine-dependent increases in spinophilin.

Term	Count	%	*p* Value	Bonferroni
mmu01200:Carbon metabolism	27	9.54	3.52 × 10^−18^	7.04 × 10^−16^
mmu05012:Parkinson’s disease	29	10.25	2.34 × 10^−17^	4.69 × 10^−15^
mmu05016:Huntington’s disease	32	11.31	9.23 × 10^−17^	2.22 × 10^−14^
mmu00190:Oxidative phosphorylation	26	9.19	4.42 × 10^−15^	8.88 × 10^−13^
mmu04721:Synaptic vesicle cycle	19	6.71	5.04 × 10^−15^	9.99 × 10^−13^
mmu00020:Citrate cycle (TCA cycle)	15	5.30	7.94 × 10^−15^	1.60 × 10^−12^
mmu05010:Alzheimer’s disease	27	9.54	1.82 × 10^−13^	3.65 × 10^−11^
mmu01130:Biosynthesis of antibiotics	29	10.25	3.85 × 10^−13^	7.70 × 10^−11^
mmu04961:Endocrine and other factor-regulated calcium reabsorption	15	5.30	1.92 × 10^−11^	3.84 × 10^−9^
mmu00010:Glycolysis/Gluconeogenesis	16	5.65	5.11 × 10^−11^	1.02 × 10^−8^

The 286 proteins that had an increased association with spinophilin (log2 ratio ≥ 0.5; Table S2A) were input into the DAVID Bioinformatics resource and analyzed using KEGG pathway analysis. The top 10 enriched pathways are shown. All pathways are given in Table S3.

**Table 5:** Top 10 GO, BP, CC, and MF pathways associated with proteins that have amphetamine-dependent increases in spinophilin. The 286 proteins that had an increased association with spinophilin (log 2 ratio ≥ 0.05; Table S2A) were input into the DAVID Bioinformatics resource and analyzed using GO BP, CC, and MF pathway analyses. The top 10 enriched pathways are shown. All pathways are given in Table S4.

Term	Count	%	PValue	Bonferroni
**Biological Process**				
GO:0006099~tricarboxylic acid cycle	13	4.59	3.85 × 10^−15^	6.78 × 10^−12^
GO:0006810~transport	68	24.03	1.55 × 10^−12^	2.70 × 10^−9^
GO:0006096~glycolytic process	11	3.89	8.12 × 10^−11^	1.42 × 10^−7^
GO:0006734~NADH metabolic process	8	2.83	1.11 × 10^−10^	1.93 × 10^−7^
GO:0046034~ATP metabolic process	11	3.89	2.57 × 10^−10^	4.48 × 10^−7^
GO:0015992~proton transport	12	4.24	7.90 × 10^−10^	1.38 × 10^−6^
GO:0015991~ATP hydrolysis coupled proton transport	10	3.53	8.81 × 10^−10^	1.54 × 10^−6^
GO:0050821~protein stabilization	15	5.30	7.11 × 10^−9^	1.24 × 10^−5^
GO:0015986~ATP synthesis coupled proton transport	8	2.83	2.98 × 10^−8^	5.20 × 10^−5^
GO:1904871~positive regulation of protein localization to Cajal body	6	2.12	3.79 × 10^−8^	6.61 × 10^−5^
**Cellular Compartment**				
GO:0043209~myelin sheath	92	32.51	3.70 × 10^−120^	1.53 × 10^−117^
GO:0070062~extracellular exosome	159	56.18	2.16 × 10^−65^	8.91 × 10^−63^
GO:0005739~mitochondrion	88	31.10	3.66 × 10^−27^	1.51 × 10^−24^
GO:0005829~cytosol	88	31.10	4.60 × 10^−26^	1.90 × 10^−23^
GO:0005737~cytoplasm	173	61.13	3.24 × 10^−22^	1.34 × 10^−19^
GO:0016020~membrane	178	62.90	6.47 × 10^−22^	2.66 × 10^−19^
GO:0014069~postsynaptic density	32	11.31	5.02 × 10^−21^	2.07 × 10^−18^
GO:0043005~neuron projection	40	14.13	6.70 × 10^−21^	2.76 × 10^−18^
GO:0043234~protein complex	46	16.25	1.63 × 10^−19^	6.71 × 10^−17^
GO:0005743~mitochondrial inner membrane	37	13.07	2.23 × 10^−19^	9.20 × 10^−17^
**Molecular Function**				
GO:0005515~protein binding	138	48.8	1.12 × 10^−22^	6.18 × 10^−20^
GO:0032403~protein complex binding	36	12.7	2.54 × 10^−18^	1.40 × 10^−15^
GO:0019901~protein kinase binding	36	12.7	1.24 × 10^−15^	6.74 × 10^−13^
GO:0044822~poly(A) RNA binding	55	19.4	4.52 × 10^−14^	2.49 × 10^−11^
GO:0000166~nucleotide binding	74	26.1	2.72 × 10^−13^	1.50 × 10^−10^
GO:0019904~protein domain specific binding	27	9.5	5.67 × 10^−13^	3.13 × 10^−10^
GO:0098641~cadherin binding involved in cell-cell adhesion	26	9.2	1.77 × 10^−12^	9.79 × 10^−10^
GO:0008022~protein C-terminus binding	22	7.8	1.30 × 10^−11^	7.15 × 10^−9^
GO:0005516~calmodulin binding	19	6.7	5.09 × 10^−10^	2.81 × 10^−7^
GO:0003779~actin binding	25	8.8	6.35 × 10^−10^	3.51 × 10^−7^

## Supplementary Materials

The Supplementary Materials are available online at http://www.mdpi.com/2227-7382/6/4/53/s1.
