# Correction: Baucum II, Anthony J. et al. Proteomic Analysis of the Spinophilin Interactome in Rodent Striatum Following Psychostimulant Sensitization. *Proteomes* 2018, *6*, 53

**DOI:** 10.3390/proteomes7010007

**Published:** 2019-02-13

**Authors:** Darryl S. Watkins, Jason D. True, Amber L. Mosley, Anthony J. Baucum

**Affiliations:** 1 Stark Neurosciences Research Institute, Indiana University School of Medicine Medical Neuroscience Graduate Program, Indianapolis, IN 46278, USA; dswatkin@iu.edu; 2 Department of Biochemistry and Molecular Biology, Indiana University School of Medicine, Indianapolis, IN 46278, USA; Jdtrue@bsu.edu (J.D.T.); almosley@iu.edu (A.L.M.); 3 Department of Biology, Ball State University, Muncie, IN 47306, USA; 4 Department of Biology, Indiana University-Purdue University Indianapolis, Indianapolis, IN 46202, USA; 5 Stark Neurosciences Research Institute Indianapolis, Indianapolis, IN 46202, USA; 6 Department of Pharmacology and Toxicology, Indiana University School of Medicine, Indianapolis, IN 46202, USA

The author wishes to make the following corrections to the methods section of their paper [1]:

The corrections include: (1) Replace the sentence “Mouse striatum (including both dorsal and ventral (accumbens) striatum or olfactory tubercle) was homogenized and sonicated in 1 mL in a low-ionic strength Tris buffer containing 20 mM Tris, 10 mM DTT, 2 mM 0.5 M EDTA, 10% Triton X-100, 1% protease inhibitor cocktail (Bimake, Houston, TX, USA)” with “Mouse striatum (including both dorsal and ventral (accumbens) striatum or olfactory tubercle) was homogenized and sonicated in 1 mL in a low-ionic strength Tris buffer containing 2 mM Tris-HCl, 1 mM DTT, 2 mM EDTA, 1% Triton X-100, and 1X protease inhibitor cocktail (Bimake, Houston, TX, USA)”; (2) replace the sentence “magnetic separation in an immunoprecipitation wash buffer (50 mM NaCl, 50 mM Tris-HCl pH 7.5, 0.5% (*v*/*v*) Triton X-100)” with “magnetic separation in an immunoprecipitation wash buffer (150 mM NaCl, 50 mM Tris-HCl pH 7.5, 0.5% (*v*/*v*) Triton X-100)”.

These corrections do not affect the conclusions of the study. We apologize for the typographical errors, and the authors would like to apologize for any inconvenience caused to the readers by these changes. The manuscript will be updated, and the original manuscript will remain online on the article webpage.
