# Supplementary material for: Proteomic Analysis of the Spinophilin Interactome in Rodent Striatum Following Psychostimulant Sensitization
Source: Proteomes. 2018 Dec 17;6(4):53. doi: 10.3390/proteomes6040053 (PMC6313900; doi:10.3390/proteomes6040053)
Supplement: Supplementary file 1 [file proteomes-06-00053-s001.zip › Figure S1_Human_top_Mouse_bottom spinophilin alignment.pdf]

```
Human  1     MMKTEPRGPGGPLRSASPHRSAYEAGIQALKPPDAPGPDEAPKGAHHKKYGSNVHRIKSM  60
             MMKTEPRGPGGPLRSASPHRSAYEAGIQALKPPDAPGPDEAPK AHHKKYGSNVHRIKSM
Mouse  1     MMKTEPRGPGGPLRSASPHRSAYEAGIQALKPPDAPGPDEAPKAAHHKKYGSNVHRIKSM  60

Human  61    FLQMGTTAGPSGEAGGGAGLAEAPRASERGVRLSLPRASSLNENVDHSALLKLGTSVSER  120
             FLQMGTTAGP GEAGGGAG+AEAPRAS+RGVRLSLPRASSLNENVDHSALLKLGTSVSER
Mouse  61    FLQMGTTAGPPGEAGGGAGMAEAPRASDRGVRLSLPRASSLNENVDHSALLKLGTSVSER  120

Human  121   VSRFDSKPAPSAQPAPPPHPPSRLQETRKLFERSAPAAAGGDKEAAARRLLRQERAGLQD  180
             VSRFDSKPAPSAQPAPPPHPPSRLQETRKLFERS PAA+GGDKEA ARRLLRQERAGLQD
Mouse  121   VSRFDSKPAPSAQPAPPPHPPSRLQETRKLFERSVPAASGGDKEAVARRLLRQERAGLQD  180

Human  181   RKLDVVVRFNGSTEALDKLDADAVSPTVSQLSAVFEKADSRTGLHRGPGLPRAAGVPQVN  240
             RKLDVVVRFNGSTEALDKLDADAVSPTVSQLSAVFEKADSRTGLHR PG PRAAG PQVN
Mouse  181   RKLDVVVRFNGSTEALDKLDADAVSPTVSQLSAVFEKADSRTGLHRAPGPPRAAGAPQVN  240

Human  241   SKLVSKRSRVFQPPPPPPPAPSGDAPAEKERCPAGQQPPQHRVAPARPPPKPREVRKIKP  300
             SKLV+KRSRVFQPPPPPP APSGD   EKER P GQQPPQHRVAPARPPPKPREVRKIKP
Mouse  241   SKLVTKRSRVFQPPPPPP-APSGDGATEKERGPGGQQPPQHRVAPARPPPKPREVRKIKP  299

Human  301   VEVEESGESEAESAPGEVIQAEVTVHAALENGSTVATAASPAPEEPKAQAAPEKEAAA-V  359
             VEVEESGESEAESAPGEVIQAEVTVHAALENGST AT ASPAPEEPKA+A PE+EAAA V
Mouse  300   VEVEESGESEAESAPGEVIQAEVTVHAALENGSTPATTASPAPEEPKAEAVPEEEAAASV  359

Human  360   APPERGVGNGRAPDVAPEEVDESKKEDFSEADLVDVSAYSGLGEDSAGSALEEDDEDDEE  419
             A  ERGV NGRAPD+APEEVDESKKEDFSEADLVDVSAYSGLGEDS GSALEEDDE+DEE
Mouse  360   ATLERGVDNGRAPDMAPEEVDESKKEDFSEADLVDVSAYSGLGEDSGGSALEEDDEEDEE  419

Human  420   DGEPPYEPESGCVEIPGLSEEEDPAPSRKIHFSTAPIQVFSTYSNEDYDRRNEDVDPMAA  479
             DGEPPYEPESGCVEIPGLSEEEDPAPSRKIHFSTAPIQVFSTYSNEDYDRRNEDVDPMAA
Mouse  420   DGEPPYEPESGCVEIPGLSEEEDPAPSRKIHFSTAPIQVFSTYSNEDYDRRNEDVDPMAA  479

Human  480   SAEYELEKRVERLELFPVELEKDSEGLGISIIGMGAGADMGLEKLGIFVKTVTEGGAAHR  539
             SAEYELEKRVERLELFPVELEKDSEGLGISIIGMGAGADMGLEKLGIFVKTVTEGGAAHR
Mouse  480   SAEYELEKRVERLELFPVELEKDSEGLGISIIGMGAGADMGLEKLGIFVKTVTEGGAAHR  539

Human  540   DGRIQVNDLLVEVDGTSLVGVTQSFAASVLRNTKGRVRFMIGRERPGEQSEVAQLIQQTL  599
             DGRIQVNDLLVEVDGTSLVGVTQSFAASVLRNTKGRVRFMIGRERPGEQSEVAQLIQQTL
Mouse  540   DGRIQVNDLLVEVDGTSLVGVTQSFAASVLRNTKGRVRFMIGRERPGEQSEVAQLIQQTL  599

Human  600   EQERWQREMMEQRYAQYGEDDEETGEYATDEDEELSPTFPGGEMAIEVFELAENEDALSP  659
             EQERWQREMMEQRYAQYGEDDEETGEYATDEDEELSPTFPGGEMAIEVFELAENEDALSP
Mouse  600   EQERWQREMMEQRYAQYGEDDEETGEYATDEDEELSPTFPGGEMAIEVFELAENEDALSP  659

Human  660   VDMEPEKLVHKFKELQIKHAVTEAEIQQLKRKLQSLEQEKGRWRVEKAQLEQSVEENKER  719
             V+MEPEKLVHKFKELQIKHAVTEAEIQQLKRKLQSLEQEKGRWRVEKAQLEQSVEENKER
Mouse  660   VEMEPEKLVHKFKELQIKHAVTEAEIQQLKRKLQSLEQEKGRWRVEKAQLEQSVEENKER  719

Human  720   MEKLEGYWGEAQSLCQAVDEHLRETQAQYQALERKYSKAKRLIKDYQQKEIEFLKKETAQ  779
             MEKLEGYWGEAQSLCQAVDEHLRETQAQYQALERKYSKAKRLIKDYQQKEIEFLKKETAQ
Mouse  720   MEKLEGYWGEAQSLCQAVDEHLRETQAQYQALERKYSKAKRLIKDYQQKEIEFLKKETAQ  779

Human  780   RRVLEESELARKEEMDKLLDKISELEGNLQTLRNSNST  817
             RRVLEESELARKEEMDKLLDKISELEGNLQTLRNSNST
Mouse  780   RRVLEESELARKEEMDKLLDKISELEGNLQTLRNSNST  817
```
