# Supplementary figures and images for: Proteomic Analysis of the Spinophilin Interactome in Rodent Striatum Following Psychostimulant Sensitization
Source: Proteomes. 2018 Dec 17;6(4):53. doi: 10.3390/proteomes6040053 (PMC6313900; doi:10.3390/proteomes6040053)

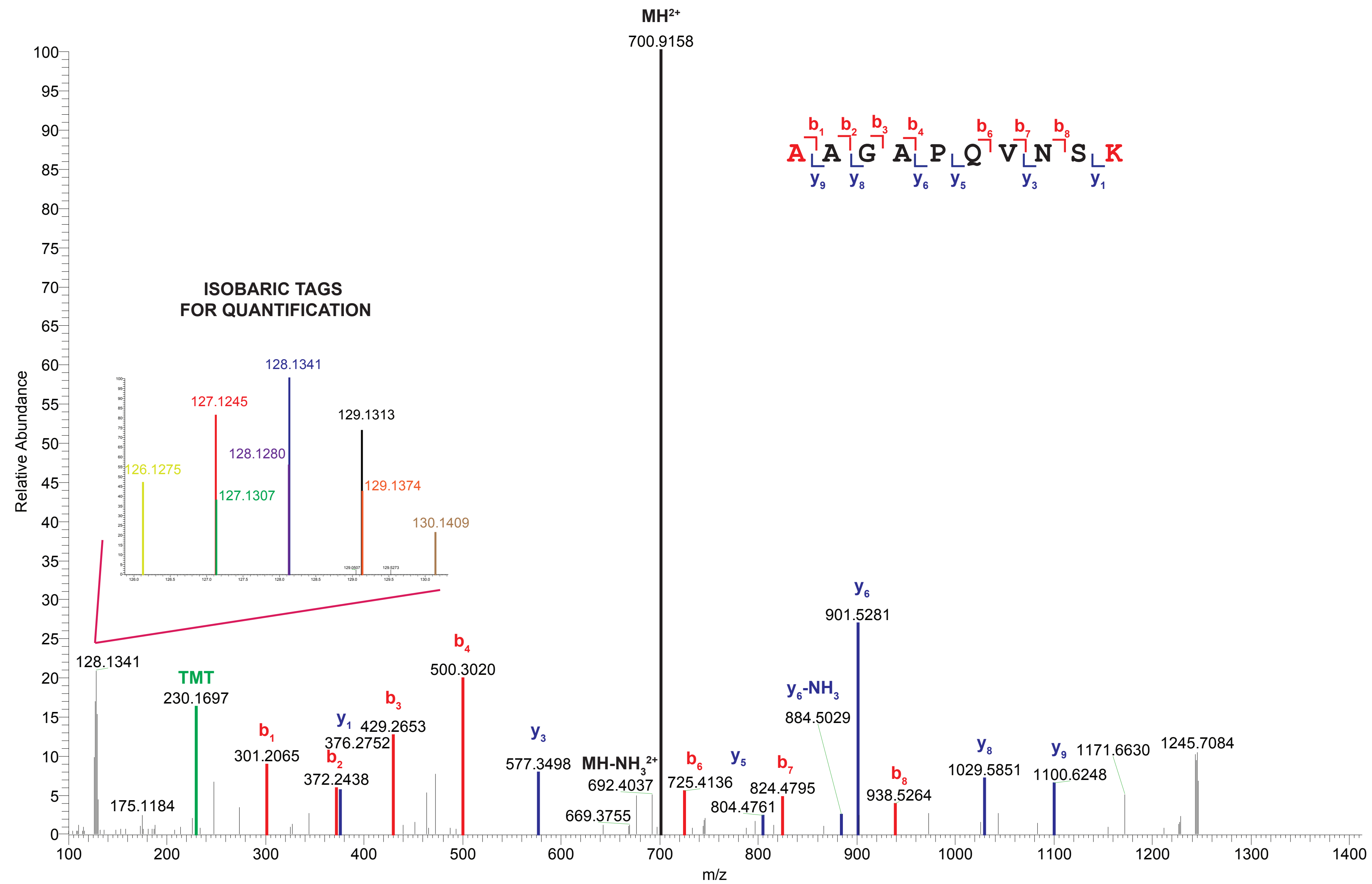

Supplement: Supplementary file 1 [file proteomes-06-00053-s001.zip › FigureS2_Mouse_AAGAPQVNSK.pdf]

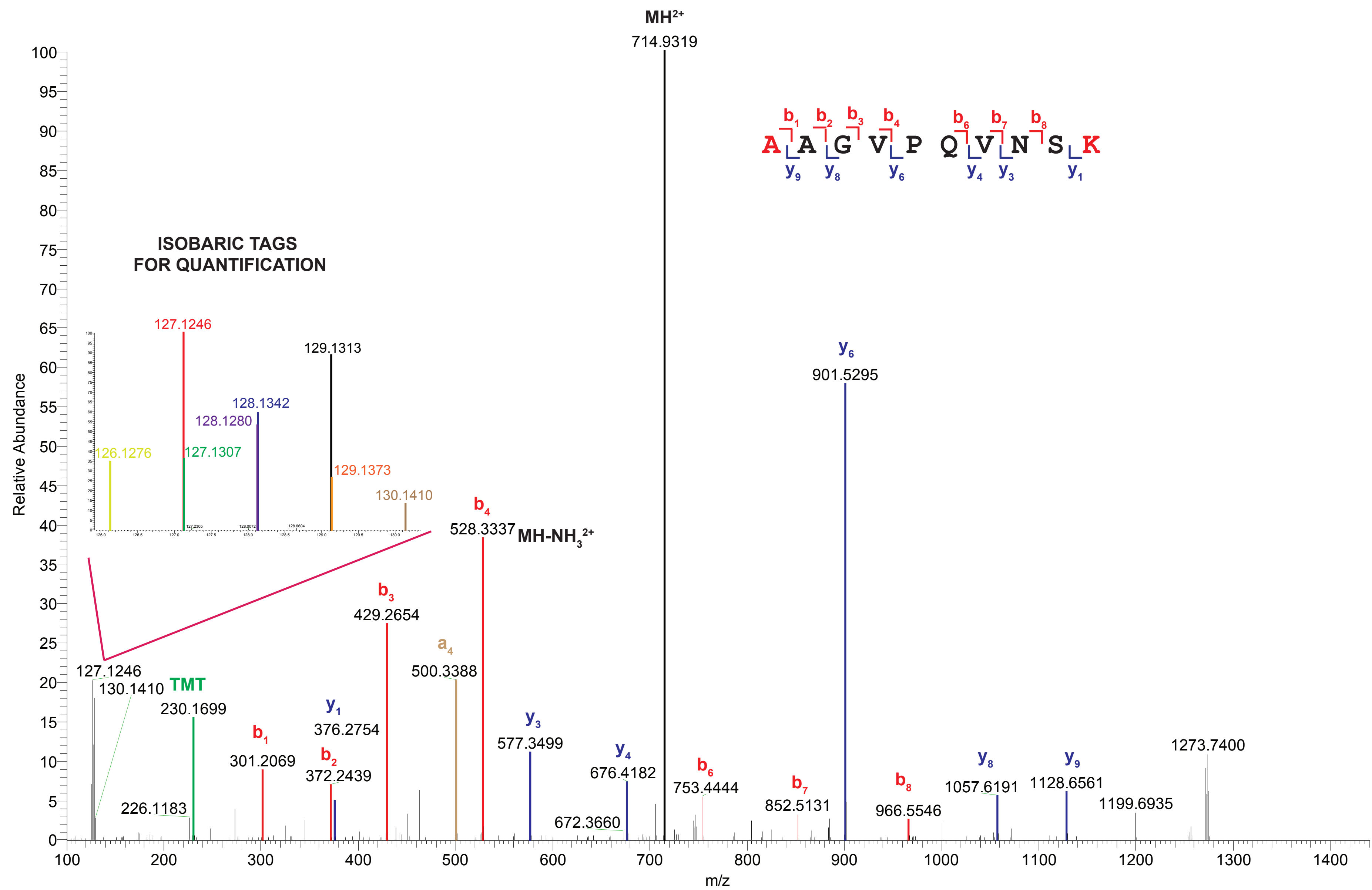

Supplement: Supplementary file 1 [file proteomes-06-00053-s001.zip › FigureS3_Human_AAGVPQVNSK.pdf]

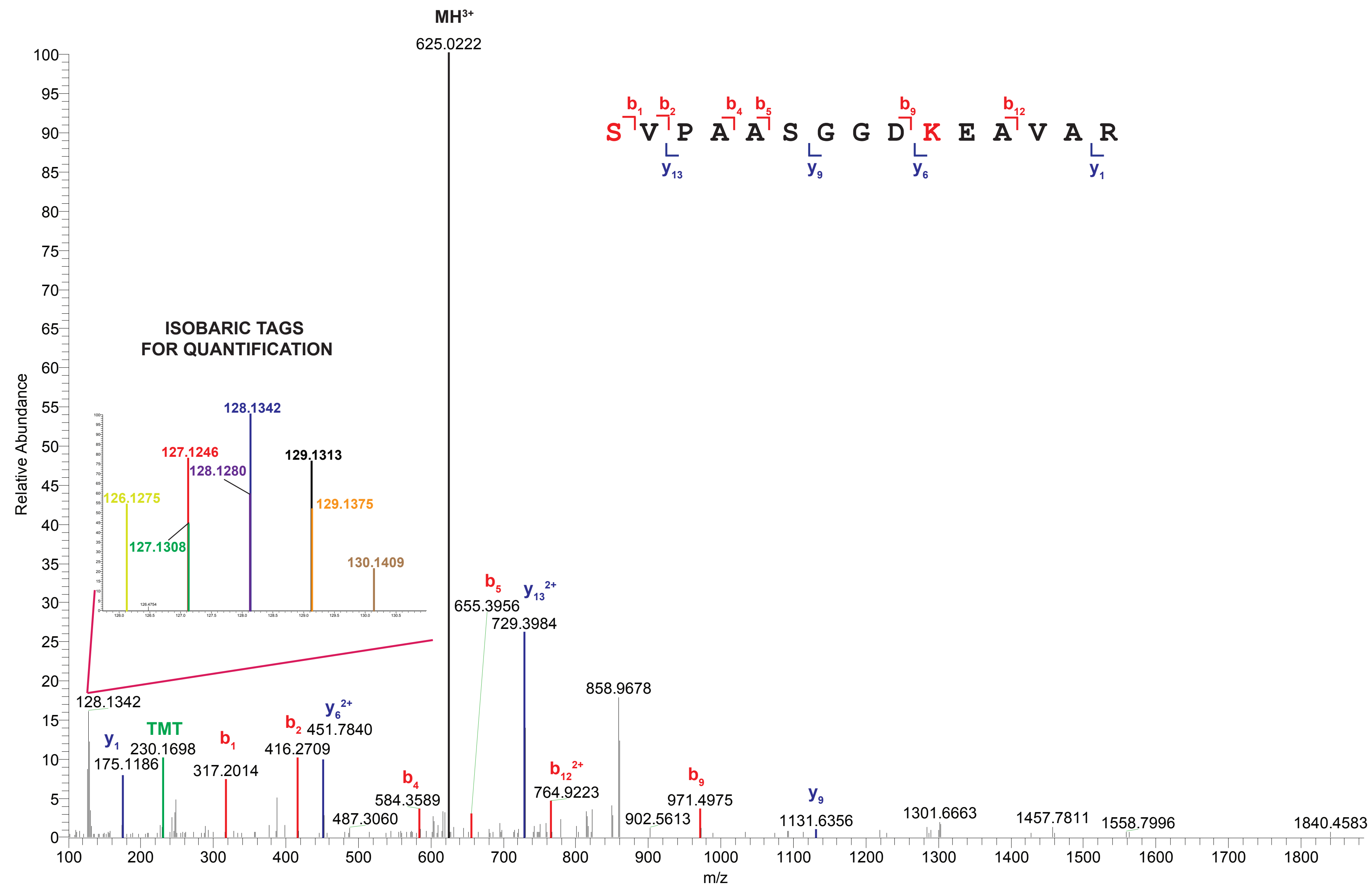

Supplement: Supplementary file 1 [file proteomes-06-00053-s001.zip › FigureS4_Mouse_SVPAASGGDKEAVAR.pdf]

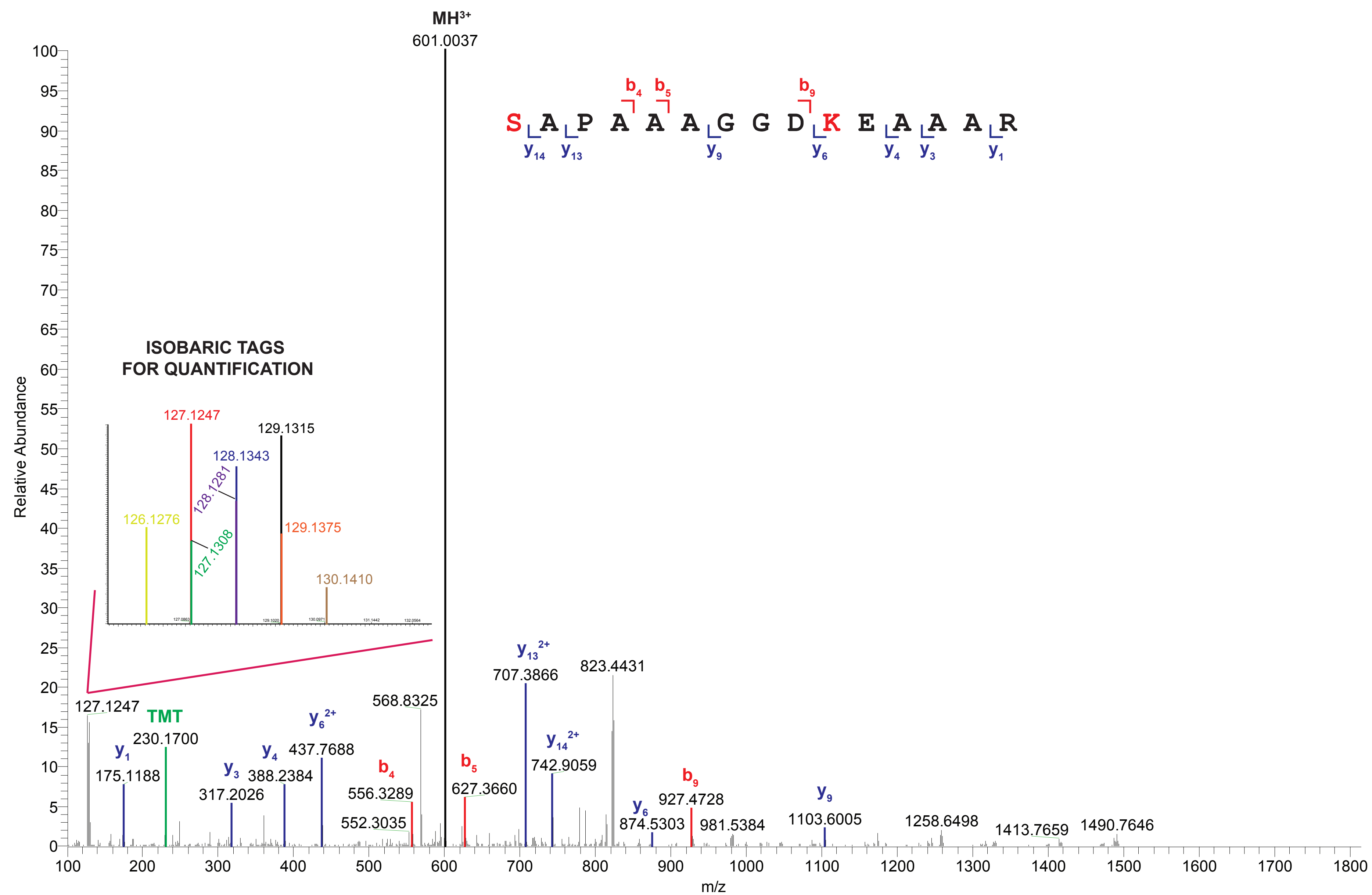

Supplement: Supplementary file 1 [file proteomes-06-00053-s001.zip › FigureS5_Human_SAPAAAGGDKEAAAR.pdf]
